# Paragangliome malin orbitaire, à propos d’un cas

**Published:** 2012-06-21

**Authors:** Idriss Benatiya Andaloussi, Mustapha Maaroufi, Mohammed Benzagmout, Taoufiq Harmouch, Meryem Abdellaoui, Salima Bhallil, Siham Tizniti, Mohammed Chaoui Elfaiz, Afaf Amarti, Hicham Tahri

**Affiliations:** 1 CHU Hassan II, Fès

**Keywords:** Paragangliome, tumeur maligne, orbite

## Abstract

Les paragangliomes sont des tumeurs neuroendocrines développées aux dépens du système nerveux parasympathique. Ils peuvent se localiser n’ importe où dans l’organisme depuis la tète et cou jusqu’au pelvis. La localisation orbitaire de cette tumeur est très rare. Nous présentons le cas d’un patient âgé de 37 ans qui présente depuis 4 mois une exophtalmie unilatérale droite, d’installation progressive, sans douleur ni baisse de l’acuité visuelle associés. L’examen général montre une tuméfaction sous le cuire chevelu, sans adénopathies locorégionales ni hépato ou splénomégalie. La tomodensitométrie retrouve un processus tumoral occupant le cadran supéro-externe de l’orbite droite, mesurant 38 mm de grand axe, envahissant la paroi supérieure et externe de l’orbite avec une importante ostéolyse. Un body scan révèle alors une métastase pulmonaire. L’examen histopathologique complétés par l’immunohistochimie, réalisé après biopsie, révèle un marquage cytoplasmique par l’anticorps anti-chromogranin, l’anticorps anti-synaptophysine et un marquage des vaisseaux par l’anticorps anti-CD31 soulignant l’architecture en zellbalen des nids tumoraux. Cet aspect est en faveur d’un paragangliome malin. Une exérèse chirurgicale incomplète suivie d’une radiothérapie adjuvante, sont alors réalisés. L’origine exacte de cette tumeur au sein de l’orbite reste très controversée. L’exophtalmie reste le principal signe révélateur. La tomodensitométrie, l’imagerie par résonnance magnétique et la scintigraphie au Metaiodobenzylguanidine radioinonisée à l’iode (MIBG-I^131^) permettent d’orienter le diagnostic et faire un bilan d’extension de la tumeur. Le diagnostic de certitude repose sur l’histopathologie et l’immunohistochimie. L’excision totale de la lésion est le traitement de choix pour les lésions bien délimitées. Dans les formes plus étendues le traitement repose sur l’excision incomplète associée à une radiothérapie adjuvante ou au MIBG I 131. La localisation orbitaire du paragangliome reste très rare. Son diagnostic est difficile et repose essentiellement sur l’immunohistochimie. Son pronostic dépend essentiellement de l’extension locale et de la présence de métastases à distance qui signe le caractère malin du paragangliome.

## Introduction

Le paragangliome, ou phéochromocytome extrasurrénalien, est une tumeur neuroendocrine développée aux dépens du système nerveux parasympathique (cellules neuroectodermiques, ou tissu paraganglionnaire). Il peut se localiser n’importe ou dans l’organisme depuis la tète et cou jusqu’au pelvis et même dans les zones ou il n’existe habituellement pas de tissus paraganglionnaire [1] ce qui peut poser des problèmes diagnostic. La localisation orbitaire de cette tumeur est très rare, le premier cas a été rapporté par Fisheret Hazard [2] et depuis lors, d’autres cas isolés ont été publié [1, 3, 4]. Nous rapportons un rare cas de paragangliome malin de l’orbite avec métastase pulmonaire, chez un homme de 37 ans, à travers lequel nous discutons les différentes caractéristiques cliniques, radiologiques, histopathologiques, immuno histochimiques et thérapeutiques de cette rare tumeur.

## Patient et observation

**Figure 1:**
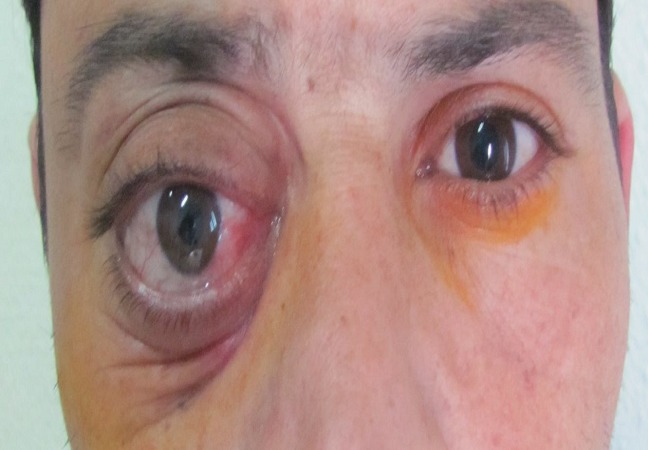
Aspect clinique du patient : exophtalmie unilatérale droite

Il s’agit d’un patient âgé de 37 ans, sans antécédents particuliers, qui présente depuis 4 mois une exophtalmie unilatérale droite, d’installation progressive, sans douleur ni baisse de l’acuité visuelle associés. L’examen ophtalmologique retrouve alors une acuité visuelle conservée à 10/10^émes^ aux deux yeux avec une exophtalmie unilatérale droite, axile, non réductible, ni pulsatile, ni soufflante et sans signes inflammatoires en regard (Figure 1). La palpation du rebord orbitaire supérieur retrouve, en profondeur, la partie antérieure d’une masse tumorale de 3 cm de grand diamètre et fixe aux plans profonds. Le reste de l’examen ophtalmologique est sans particularités. L’examen général montre une tuméfaction sous le cuire chevelu, ferme et fixe aux plans profonds, sans adénopathies locorégionales ni hépato ou splénomégalie.

**Figure 2:**
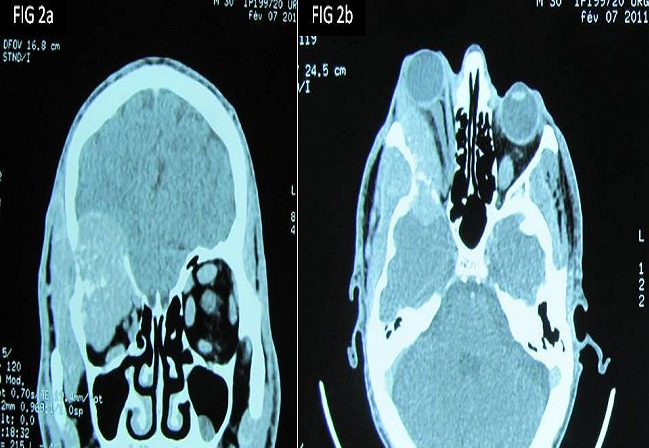
Aspect tomodensitométrique de la tumeur qui envahit la paroi supérieure et externe de l’orbite avec une importante ostéolyse et une réaction périostée en feu d’herbe. 2a : coupe coronale, 2b coupe axiale

**Figure 3:**
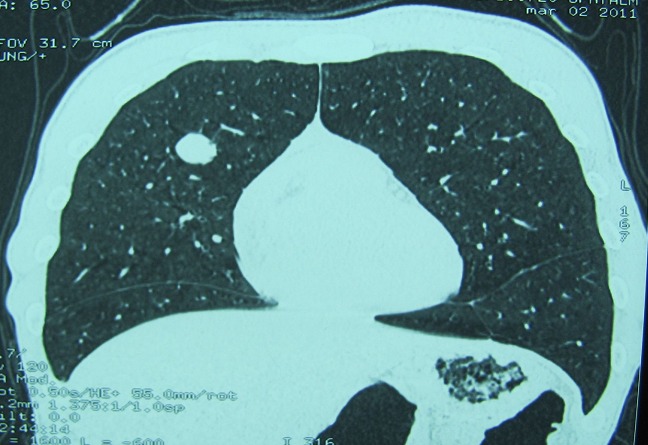
Aspect tomodensitométrique révélant une métastase pulmonaire droite

**Figure 4:**
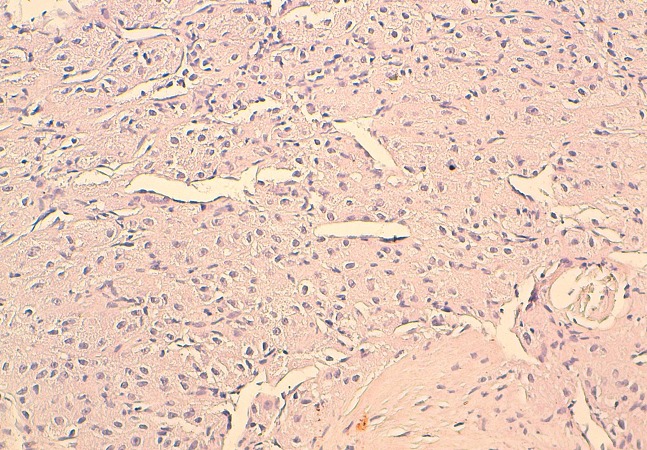
Examen histopathologique (HES ×200): prolifération tumorale montrant une vascularisation abondante et fine avec des cellules tumorales globuleuses à cytoplasme abondant et éosinophile et un noyau régulier

**Figure 5:**
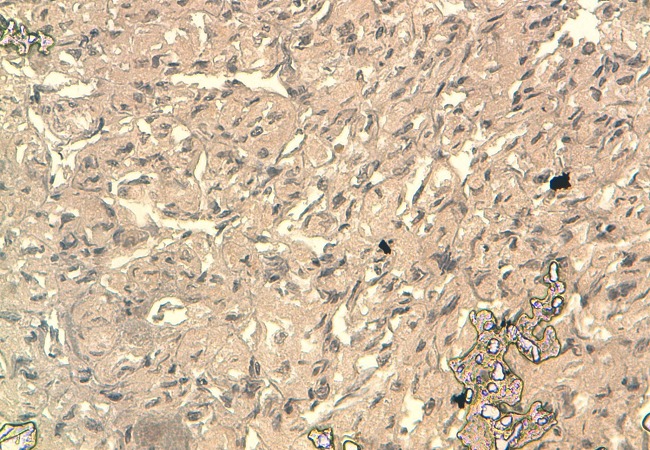
Immunohistochimie : marquage cytoplasmique par l’anticorps anti-chromogranin

**Figure 6:**
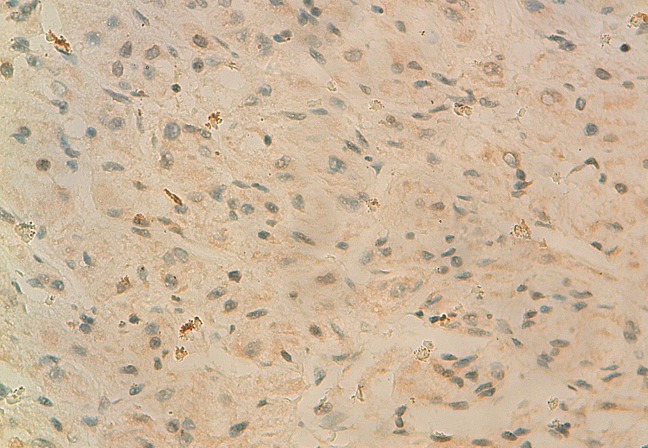
Immunohistochimie: marquage cytoplasmique par l’anticorps anti-synaptophysine

**Figure 7:**
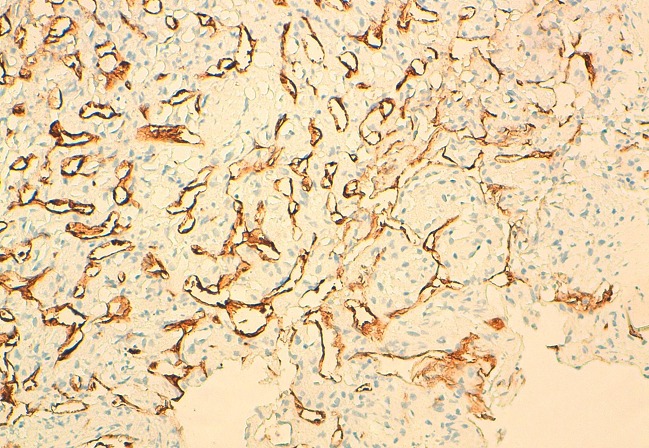
Immunohistochimie (×200): marquage des vaisseaux par l’anticorps anti-CD34 soulignant l’architecture en zellbalen des nids tumoraux

La tomodensitométrie retrouve un processus tumoral occupant le cadran supéro-externe de l’orbite droite, mesurant 38 mm de grand axe, spontanément dense et rehaussé après injection du produit de contraste. Ce processus envahit la paroi supérieure et externe de l’orbite avec une importante ostéolyse et une réaction périostée en feu d’herbe. Il s’étend en haut vers le lobe frontal, en dehors vers la fosse temporale et en dedans en intraorbitaire envahissant la glande lacrymale et les muscles droits supérieurs et externes (Figure 2 (a et b)). Une autre lésion ostéolytique avec les mêmes caractéristiques est présente au niveau de la voute occipitale envahissant le sinus longitudinal supérieur. Un body scan révèle alors une métastase pulmonaire ((Figure 3). L’examen histopathologique, réalisé après biopsie, montre une prolifération tumorale montrant une vascularisation abondante et fine avec des cellules tumorales globuleuses à cytoplasme abondant et éosinophile et un noyau régulier ((Figure 4). L’immunohistochimie révèle un marquage cytoplasmique par l’anticorps anti-chromogranin ((Figure 5), l’anticorps anti-synaptophysine ((Figure 6) et un marquage des vaisseaux par l’anticorps anti-CD31 et anti-CD34 soulignant l′architecture en zellbalen des nids tumoraux ((Figure 7). Cet aspect est en faveur d’un paragangliome malin.

Devant l’extension locale et générale de la tumeur, nous avons procédé à une embolisation artérielle des branches de l’artère carotide externe destinées à la tumeur afin de réduire le risque hémorragique préopératoire, puis 48 heurs après à une exérèse chirurgicale incomplète par craniotomie. L’évolution est marquée par une régression de l’exophtalmie. Une radiothérapie adjuvant est alors démarrée.

## Discussion

La localisation orbitaire du paragangliome reste exceptionnelle puisqu’à ce jour seulement 36 cas ont été publié depuis la première description faite par Fisher et Hazard en 1952 [1–4]. L’origine exacte de cette tumeur au sein de l’orbite reste très controversée en l’absence de description à ce jour de tissu paraganglionnaire intra orbitaire chez l’être humain. Pour certain auteur cette prolifération proviendrait du tissu sustentaculaire ou du ganglion ciliaire [3].

L’âge de survenue des paragangliomes orbitaires est très variable allant de 3,5 à 68 ans (moyenne 32,8 ans) sans prédominance d’un sexe par rapport à l’autre. La durée des symptômes varie de 2 mois à 17 ans. La plupart des cas se présentent avec exophtalmie d’allure tumorale. D’autres symptomes sont également rapportés: diplopie, oedème papillaire, et diminution de l’acuité visuelle [3].

A la tomodensitométrie, ces lésions sont bien définies, homogènes, isodenses et uniformément rehaussées. L′IRM montre une prédominace des vaisseaux sanguins sous la forme d’absence de signal avec un aspect poivre et sels [3, 4]. En dehors de la présence des métastases à distance, il n′est pas possible de différencier avec l′imagerie seule le caractère bénin ou malin des paragangliomes. Toutefois, certaines caractéristiques peuvent orienter vers le caractère malin par la présence d’une taille élevée, une nécrose confluente, une invasion vasculaire ou une large extension locale [5].

Le Metaiodobenzylguanidine (MIBG) est un analogue de la norépinephrine qui se concentre au niveau de la médullo-surrénale et de certaines tumeurs neuro-ectodermiques comme le paragangliome. La scintigraphie au MIBG radioionisé à l’iode 131 ou 123 permet une détection plus précise des paragangliomes et des différents sites métastatiques avec une sensibilité qui atteint 67 à 100% [6].

La confirmation du diagnostic de paragangliome repose sur l’examen anatomo-pathologique complété par l’étude immunohistochimique. En effet, morphologiquement, ces lésions sont caractérisées par un aspect alvéolaire ou de nidification (Zell Ballen) Ces nids de cellules à cytoplasme granulaire sont séparés par des cloisons fibrovasculaire minces et accentuées par la coloration à l′argent pour la réticuline. Sur le plan immunohistochimique, la paragangliome est caractérisé par une positivité à la synaptophysine et la chromogranine [1, 3, 4].

Les principaux diagnostics différentiels des paragangliomes comprennent: 1) le sarcome alvéolaire des parties molles qui est éliminé devant la présence des granules PAS positifs ou des cristaux en forme d’aiguille et l’immunonégativité à la synaptophysine et la chromogranine [3]; 2) le rhabdomyosarcome alvéolaire est caractérisé par des cellules de plus petites taille, et une immunohistochimie positive pour l′actine, la myoglobine, la desmine, et myo-D [3, 4, 7]; 3) le neuroblastome orbitaire et les carcinoïdes peuvent être exclues sur la base de caractéristiques morphologiques et immunohistochimiques.

Généralement, les paragangliomes sont plus agressifs que les phéochromocytomes. La diffusion se fait par voie hématogène et lymphatique, avec des métastases qui siègent préférentiellement au niveau des ganglions lymphatiques régionaux, l’os, le foie et le poumon [5].

L′excision totale de la lésion, grâce à l’orbitotomie latéral, est le traitement de choix pour les lésions bien délimitées. Toutefois, l′excision incomplète est recommandée pour une lésion en contact avec le nerf optique. L’exentération orbitaire doit être réservée aux tumeurs de croissance rapide, incomplètement excisées ou récurrentes [3]. La radiothérapie est utile pour arrêter la croissance d′une tumeur à croissance rapide avant la chirurgie ou comme une modalité alternative en cas d’impossibilité d’exérèse chirurgicale complète en raison de la taille et/ou de l’extension de cette tumeur [3, 7].

Le MIBG radioionisé à l’iode 131 peut être utilisé non seulement à visée diagnostic mais aussi à visée thérapeutique dans les formes matastatiques des paragangliomes malins avec une nette amélioration de la survie à 5ans passant de 44% à 65% [8].

Les paragangliomes sont rarement malins et l′atteinte multifocale survient dans 10% à 20% des cas, surtout s′il ya prédisposition familiale [3, 7]. L’évolution est marquée par le développement d’une deuxième lésion dans 5% à 10% [3, 7]. Rarement, le paragangliome intracrânien peut s′étendre sur une orbite et provoquer une exophtalmie [3, 9, 10].

## Conclusion

La localisation orbitaire du paragangliome reste très rare. Son diagnostic est difficile et repose essentiellement sur l’immunohistochimie. Son pronostic dépend essentiellement de l’extension locale et de la présence de métastases à distance qui signe le caractère malin du paragangliome.
